# An All Fiber White Light Interferometric Absolute Temperature Measurement System

**DOI:** 10.3390/s8116825

**Published:** 2008-11-01

**Authors:** Jeonggon Harrison Kim

**Affiliations:** Information and Communication Engineering Dept. Hansei University, 604-5 Dangjeong-dong Gunpo-city Kyunggi-do, Korea; Tel. 82-2-31-450-5174; Fax. 82-2-31-450-5172; E-mail:jeongkim@hansei.ac.kr

**Keywords:** All fiber white light interferometry, signal processing algorithm, absolute temperature measurement

## Abstract

Recently the author of this article proposed a new signal processing algorithm for an all fiber white light interferometer. In this article, an all fiber white light interferometric absolute temperature measurement system is presented using the previously proposed signal processing algorithm. Stability and absolute temperature measurement were demonstrated. These two tests demonstrated the feasibility of absolute temperature measurement with an accuracy of 0.015 fringe and 0.0005 fringe, respectively. A hysteresis test from 373K to 873K was also presented. Finally, robustness of the sensor system towards laser diode temperature drift, AFMZI temperature drift and PZT non-linearity was demonstrated.

## 1. Introduction

In order to fully utilize the capability of fiber optic sensors, a fiber optic sensor with a new sensing principle termed as “White Light Interferometry” (WLI) was developed [1]. A white light interferometer differs from a conventional one in that it uses a broadband light source and two interferometers are connected in tandem. These two interferometers are the sensing interferometer and the processing interferometer (scanning interferometer). For the case of white light interferometry, the zero order fringe peak (central fringe) is the peak with the highest fringe visibility amplitude and the absolute Optical Path Difference (OPD) of the sensing interferometer is known if the zero order fringe peak is identified. White light interferometry has the potential to identify the interference fringe order from the output pattern (fringe scan) of an interferometer [2], but for most broadband light sources, the visibility of fringes varies so slowly in the vicinity of the zero order fringe peak that a high signal-to-noise ratio (SNR) is required to identify the zero order fringe peak simply through the inspection of its magnitude. This difficulty has inhibited the application of fiber optic sensors using WLI for absolute OPD determination [3]. Recently, the author proposed a new signal processing algorithm (hereafter signal processing algorithm) for an all fiber white light interferometry and the proposed signal processing algorithm has been proven to be effective for absolute temperature measurement. Computer simulations of the proposed signal processing algorithm identified the zero order fringe peak with a miss rate of 0.0003 at 31 [dB] signal-to-shot noise ratio. Also, at 35 [dB] signal-to-shot noise ratio, a resolution of 10^−3^ fringe was obtained. This article proposes an all fiber white light interferometric absolute temperature measurement system using the previously proposed signal processing algorithm and demonstrates a stability test, an absolute measurement test and a hysteresis test. This article also demonstrates the robustness of the proposed sensor system.

## 2. Previous Works

Two kinds of well-known distinct methods for the observation of white light interferometric fringe patterns in optical fiber sensor systems are spectral domain processing and phase domain processing [4]. Phase domain processing method can be further divided into two classes, depending on the method of pattern formation, namely temporal fringe formation and spatial fringe formation.

In the spectral domain processing, a spectrum analyzer is used to directly process the output of sensing interferometer with a spectral resolution capability of Δ*λ* [nm ]. Any temperature change affecting the sensing interferometer induces a change in modulation frequency of the measurement signal and the position of maxima (fringe peaks) in the spectral pattern as well, which can be measured and mapped to the temperature. Reference [5] proposed a WLI temperature sensor system where a low finesse reflective mode Fabry-Perot interferometer is used as a sensing interferometer and an optical spectrum analyzer is used as a processing unit. This WLI system attained (absolute) temperature measurement over the range from 298K to 673K with resolution equal to 1 K, but, the relation between temperature and modulation frequency (fringe spacing of the spectrum of output of sensing interferometer) was not quite linear and it is questionable to apply the result for absolute temperature measurement. Two major issues of the current spectral domain techniques are the slow response, which limits them to the measurement of static or quasi-static signals such as temperature, displacement, strain, pressure and the requirements of expensive spectrum processing components such as diffraction gratings and detector arrays, especially those operating in the 1.0∼1.7 [μm] band. For example, a spectrometer at the visible band may cost more than $2,000, while one at 1.55 [μm] may cost ten times higher [6].

In spatial fringe formation, the scanning of the OPD of the processing interferometer is achieved by the use of a charge-coupled-device (CCD) array in order to detect the spatial interference fringes formed in the so-called “electronically-scanned” arrangement [7]. Reference [7, 3] used a Michelson interferometer with one of its two mirrors being slightly tilted to form the spatially distributed interference fringes. Computer simulations of reference [3] showed that the misidentification rate of central fringe was almost “zero” and the resolution of 1/400 fringe was obtained at 26 [dB] SNR. Experiments in reference [7] have demonstrated a repeatability of 0.02K within a 1K temperature range and the repeatability of 0.6K within a 100K temperature range for absolute temperature measurement. One of the main difficulties with the use of these configurations is the so-called spatial “mis-overlapping ' of the beams, i.e. the cross-section of one beam cannot be exactly overlapped on that of the other [7]. As a result, the effective scanning range of the system will be reduced over what may otherwise be achieved.

In temporal fringe formation, the adjustment of the OPD of the processing interferometer may be achieved by the use of sophisticated methods such as a piezoelectric transducer (PZT) to produce a “mechanically-scanned” interferometer [8] and OPD of the processing interferometer is scanned to match to that of the sensing interferometer. Since the measurement is achieved by the comparison of the values of the OPDs of the two interferometers, this technique is immune to wavelength and power fluctuation induced noise. This technique also yields a high resolution and a large dynamic range [9]. Reference [10] showed that the fringe visibility out of white light sensor system, comprised of a sensing FFPI operating in the reflective mode and a reference FFPI operating in the transmissive mode, depends on the sensor (absolute) temperature. One drawback, however, is that fringe visibility is varying along the envelope of interferogram and fringe visibility gets reduced towards the outer region of interferogram. This can limit the temperature range of operation, and thus be a drawback in some applications. Reference [11] proposed a white light interferometer using a bulk Michelson interferometer and two FFPIs (sensing FFPI and reference FFPI) and reported that 0.0025 fringe (0.013 K) of accuracy and dynamic range of room temperature to 1073K were obtained. This reference showed the feasibility of white light interferometer absolute temperature which is converted into the difference in the OPDs of the reference interferometer and the sensing interferometer, but this approach suffers from the fact that the light is guided out of the fiber into a bulk optic system. This introduces losses and makes the system more sensitive to external perturbations such as vibration. One approach to overcome these problems is to use an all fiber system for performing both the sensing and scanning processes. The light would then be confined to the fiber reducing losses and would be robust to the external environment comparing to the bulk optic systems. Interested users are referred to references [24-27] for more information about fiber optic temperature sensor based on white light interferometric technique.

This article proposes an all fiber white light interferometric system providing PZT-based optical path scanning, demonstrated in conjunction with absolute temperature measurements. Results of the stability test, the absolute temperature measurement test and the hysteresis test are presented characterizing the performance of the proposed all fiber white light interferometric absolute temperature measurements.

## 3. All fiber white light interferometry

**Figure 1.**
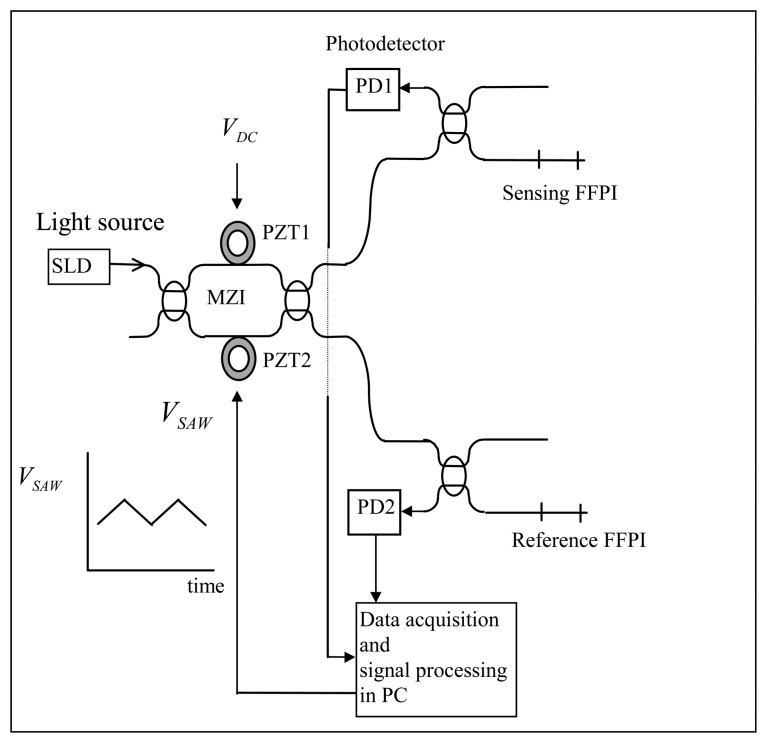
All Fiber White Light Interferometer.

This research proposes the high resolution absolute temperature measurement system using white light interferometry. Figure 1 shows the experimental setup for an All Fiber White Light Interferometry (hereafter AFWLI). Experimental absolute temperature measurement system consists of three interferometers: the sensing interferometer, the reference interferometer, and the processing interferometer. Fiber Fabry-Perot interferometer (FFPI) is a sensing interferometer and also a reference interferometer. The FFPIs are composed of two TiO_2_ thin film internal mirrors [12] that forms a cavity ∼10 mm long. An All Fiber Mach-Zehnder Interferometer (hereafter AFMZI) was used as a processing interferometer and approximately 30 meters of single mode fiber was wrapped in two layers round the Piezoelectric Transducer Tubes (hereafter PZT) inserted in the scanning arms of MZI. Only moving parts employed were two PZTs on scanning arms of AFMZI. Two photodetector outputs (PD1 and PD2 in Figure 1) were sampled, digitized and stored using a data acquisition board. The light source used in Figure 1 was an Oki OE350S (*λ* = 1.3 μm). Superluminescent Diode (SLD) of Oki Semiconductor with coherence length *L_C_* ≈ 26*λ*.

The light source SLD package includes an internal thermoelectric cooler (TEC) and an internal thermistor. Feedback temperature control using a TEC and a thermistor is used to maintain a constant temperature of light source and to minimize the dependence of sensor system's accuracy on the light source temperature drift. Also, AFMZI and two PZTs were placed in the temperature-stabilized double copper chamber which has air gap between outer chamber and inner chamber. Double copper chamber was equipped with one TEC and two thermistors (one for monitoring and one for temperature control). Temperature of double copper chamber was controlled using a TEC and a thermistor so that we can minimize the effect of environmental temperature on PZTs and AFMZI.

## 4. Principle of absolute temperature measurement

**Figure 2.**
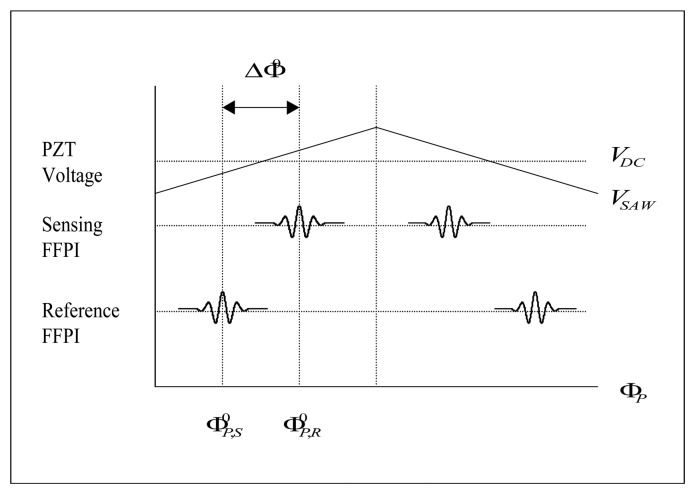

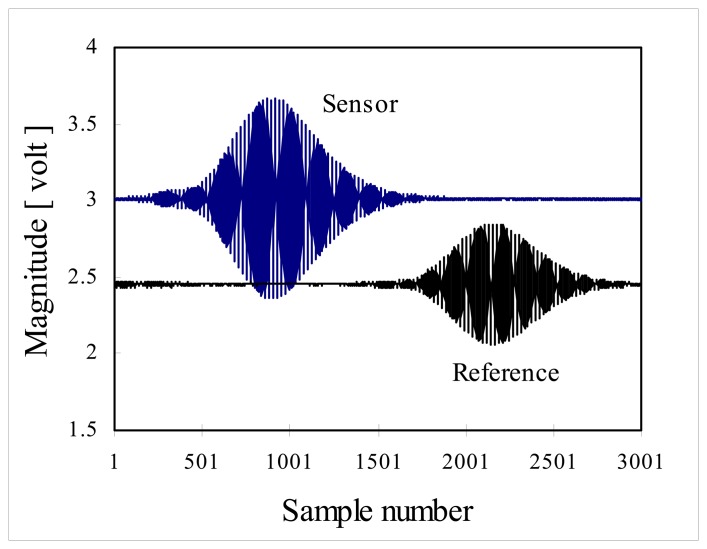
**a.** Outputs (fringe scans) of sensing and reference FFPI. **b.** Typical sensor and reference fringe scan.

The proposed AFWLI consists of three interferometers, one processing interferometer (AFMZI) and two FFPIs. One FFPI is working as a sensing interferometer exposed to the temperature *T_S_* to be measured and the other FFPI is working as a reference interferometer which is protected from environmental disturbances but exposed to the known reference temperature *T_R_*. In this set-up the AFMZI is scanned to match the phase of the MZI to that of the sensing FFPI, such that AFMZI and sensing FFPI are coherently matched and interfered. The processing AFMZI has two PZTs in its two arms. A constant D.C. voltage *V_DC_* (100∼150 [volt], Figure 1 and Figure 2a) is applied to the PZT1 in one arm to coarsely match the OPD of AFMZI to that of the sensing FFPI within the coherence length of the light source. An alternating ramp voltage *V_SAW_* (Figure 2a) is applied to PZT2 in the other arm to scan the processing interferometer so that the zero order fringe peak for each FFPI occurs when its round-trip OPD exactly matches the OPD of the MZI.

The sensing and the reference interferometer outputs (fringe scans) of AFWLI are given by:

(1) $$I_{s}\left(\Phi_{P}\right)=1+\frac{1}{2}\exp\left\{-{\left[\frac{\Phi_{P}-\Phi_{S}}{\pi L_{C}/\lambda }\right]}^{2}\right\}\cos\left(\Phi_{P}-\Phi_{S}\right)$$

(2) $$I_{R}\left(\Phi_{P}\right)=1+\frac{1}{2}\exp\left\{-{\left[\frac{\Phi_{P}-\Phi_{R}}{\pi L_{C}/\lambda }\right]}^{2}\right\}\cos\left(\Phi_{P}-\Phi_{R}\right)$$

Typical sensor and reference fringe scan are shown in Figure 2b. In Eq. (1) and (2), the OPD Φ*_P_* of the AFMZI, the optical phase differences induced by the sensing FFPI (the phase difference to be measured) and the reference FFPI are given by $\Phi_{P}=\frac{2\pi nL_{P}}{\lambda }$, $\Phi_{S}=\frac{2\pi nL_{S}}{\lambda }$, and $\Phi_{R}=\frac{2\pi nL_{R}}{\lambda }$, respectively, where *L_P_* is the path length of AFMZI, *L_S_* and *L_R_* are the round trip path difference of the sensing FFPI and the reference FFPI respectively, *n* is the refractive index of the optical fiber core. Now, assume that the known temperature of the sensing FFPI and the reference FFPI are $T_{S}^{0}$, $T_{R}^{0}$ respectively. When the OPD of AFMZI, Φ*_p_* is exactly matched to that of sensing FFPI, output of sensing FFPI is maximized (zero-order fringe peak) at certain $\Phi_{P,S}^{0}$ (Figure. 2a and 2b) where

(3) $$\Phi_{P,S}^{0}=\frac{2\pi nL_{S}}{\lambda }$$

Following the same token, the reference FFPI will give its own zero order fringe peak at certain $\Phi_{P,R}^{0}$ (Figure. 2a and 2b) where

(4) $$\Phi_{P,R}^{0}=\frac{2\pi nL_{R}}{\lambda }$$

Then, we can get the known value of (absolute) phase difference $\Delta \Phi^{0}=\Phi_{P,S}^{0}-\Phi_{P,R}^{0}$ (this is possible by the proposed signal processing algorithm in Section 5) and ΔΦ^0^ is mapped (calibrated) to the temperature $T_{S}^{0}$.

When a sensing FFPI is exposed to another temperature $T_{S}^{i}$, phase change $\Delta \Phi_{S}^{i}$ induced by the temperature shift $\Delta T_{S}^{i}=T_{S}^{i}-T_{S}^{0}$ of sensing FFPI is given by [13]:

(5) $$\Delta \Phi_{S}^{i}=\frac{2\pi }{\lambda }\left(\frac{n}{L_{S}}\frac{dL_{S}}{dT}+\frac{dn}{dT}\right)\Delta T_{S}^{i}L_{S}$$

neglecting the temperature-induced change in the optical fiber diameter. Then the phase of processing interferometer will have its zero order fringe peak at:

(6) $$\Phi_{P,S}^{i}=\Phi_{P,S}^{0}-\Delta \Phi_{S}^{i}$$

But phase $\Phi_{P,R}^{i}=\Phi_{P,R}^{0}$ as the reference interferometer is protected from the environmental disturbances and phase delay

(7) $$\Delta \Phi^{i}=\Phi_{P,S}^{i}-\Phi_{P,R}^{i}=\Phi_{P,S}^{i}-\Phi_{P,R}^{0}$$

is mapped (calibrated) to temperature $T_{S}^{i}$. By repeating this procedures for the other temperatures $T_{S}^{j}$, all the ΔΦ*^j^* 's are mapped to the all the sensing FFPI temperatures $T_{S}^{j}$.

At this point, we need to check if mapping ΔΦ*^j^* to $T_{S}^{j}$ is uniquely determined. Applying same assumption mentioned above for Eq. (5) the phase change $\Delta \Phi_{P}^{i}$ induced by axial-straining the fiber on the PZT can be given by [14]

(8) $$\Delta \Phi_{P,S}^{i}=\frac{2\pi }{\lambda }\left(\frac{n}{L_{P}}+\frac{dn}{dL_{P}}\right)\Delta L_{P}L_{P}$$

But note that $\Phi_{P,S}^{i}$ in Eq. (6) can be also expressed as

(9) $$\Phi_{P,S}^{i}=\Phi_{P,S}^{0}+\Delta \Phi_{P,S}^{i}$$

because the phase change $\Delta \Phi_{S}^{i}$ in Eq. (6) is compensated by the phase change $\Delta \Phi_{P,S}^{i}$ in Eq. (9), which is induced by scanning AFMZI. So we can equate these two terms, $\Delta \Phi_{S}^{i}$ of Eq. (5) and $\Delta \Phi_{P,S}^{i}$ of Eq. (8) by considering only dominant terms [14]:

(10) $$\frac{2\pi }{\lambda }\left(\frac{dn}{dT}\right)\Delta T_{S}^{i}L_{S}=\frac{2\pi }{\lambda }\left(\frac{n}{L_{P}}\right)\Delta L_{P}L_{P}$$

which results in:

(11) $$\Delta L_{P}=\frac{dn}{dT}\frac{L_{S}}{n}\Delta T_{S}^{i}$$

From Eq. (11) it is shown that temperature change $\Delta T_{S}^{i}$ in the sensing FFPI has a linear (one-to-one) relation with the length change Δ*L_P_* in the processing interferometer. Again, ΔΦ*^i^* is given as

(12) $$\Delta \Phi^{i}=\Phi_{P,S}^{i}-\Phi_{P,R}^{0}=\Phi_{P,S}^{0}+\Delta \Phi_{P,S}^{i}-\Phi_{P,R}^{0}$$

$\Phi_{P,S}^{0}$ and $\Phi_{P,R}^{0}$ in Eq. (12) are fixed constant phase delay at temperature $T_{S}^{0}$, $T_{R}^{0}$ and $\Delta \Phi_{P,S}^{i}(\text{or}\Delta L_{P})$ in Eq. (12) is linearly related to $\Delta \Phi_{S}^{i}(\text{or}\Delta T_{S}^{i})$. Then, at any temperature $T_{S}^{i}$, the phase delay ΔΦ*^i^* between the reference FFPI and the sensing FFPI is linearly and uniquely mapped to the absolute temperature $\Delta T_{S}^{i}$ over the range in which linearity assumed in Eq. (5) and Eq. (8) is effective.

This kind of sensor is self-calibrating and is capable of absolute temperature measurement. Also this kind of sensor is not limited by the quadrature stabilization and fringe counting problem. Another desirable feature is the large dynamic range, while a limitation is that the reference interferometer has to be kept independent of the measurand. The dynamic range of the measurement depends on the dynamic range of the sensing FFPI and scanning MZI. A FFPI temperature sensor with internal mirror was tested from 73K to 1323 K [15]. And using high voltage PZTs total scanning range of 150 μm is possible [16] which approximately corresponds to 115 fringes or 963 K temperature change for FFPI sensor of 10mm cavity length.

## 5. Digital Signal Processing Algorithm

The proposed signal processing algorithm calculates the phase delay ΔΦ*^i^* of OPDs of the sensing FFPI and the reference FFPI by calculating the position of the zero order fringe peak in the cross-correlation fringe of the sensing FFPI fringe scan and the reference FFPI fringe scan. As a preliminary procedure, the outputs of photodetector signals *I_R_*(Φ*_P_*) and *I_S_*(Φ*_P_*) are sampled and stored in fringe scan *I_S_*(*n*) and *I_R_*(*n*), respectively. *I_S_*(*n*) and *I_R_*(*n*) are cross-correlated into *i*(*n*) (sample number index *n* should not be confused with refractive index *n*). Then any D. C. component *i*(*n*) is removed and of all the peaks *p_i_* of *i*(*n*) are registered. The proposed signal processing algorithm is based on the hypothesis test in which algorithm selects nine biggest peaks *p_j_* (*j*=0,±1,±2,±3±4) as the zero order fringe peak candidates and calculates the parameter *g*(*p_j_*) for each and every zero order fringe peak candidate:

(13) $$g(p_{j})=\sum_{i=0}^{\infty }\left\{i(p_{j+i+1})-i(p_{j-i-1})\right\}=\sum_{i=0}^{\infty }d(j,i)$$

where *p*_1_ is the positive first order fringe peak, *p*-_1_ is the negative first order fringe peak and so on. In other words, hypothesis test presumes each candidate peak is the zero order fringe peak and calculates the parameter *g*(*p_j_*) in Eq. (13). Then, for zero order fringe peak *p*_0_*g*(*p*_0_) is given as:

(14) $$g(p_{0})=\sum_{i=0}^{\infty }\left\{i(p_{i+1})-i(p_{-i-1})\right\}=\left\{i(p_{1})-i(p_{-1})\right\}+\left\{i(p_{2})-i(p_{-2})\right\}+\left\{i(p_{3})-i(p_{-3})\right\}+\ldots .$$

which must be zero because ideally all the values of *d*(*j,i*) for the zero order fringe peak (*p*_0_, *j* = 0) are zero due to the symmetry property of *i*(*p_i_*_+1_) = *i*(*p*_−_*_i_*_−1_) . Ideally all the entries of *d*(*j,i*) for all *j* except *j* = 0 are non-zero terms and hypothesis test simply must look for any zero order fringe peak candidate with *g*(*p_j_*) = 0 . This is the same condition as the hypothesis test looks for zero order fringe peak candidate about which *i*(*n*) (or envelope of *i*(*n*)) is symmetric. But, practically the cross-correlation function *i*(*n*) is not perfectly symmetric with respect to the zero order fringe peak due to the noise in fringe scans and any zero order fringe peak candidate with the smallest |*g*(*p_j_*)| is announced as the zero order fringe peak in the hypothesis test.

**Figure 3.**
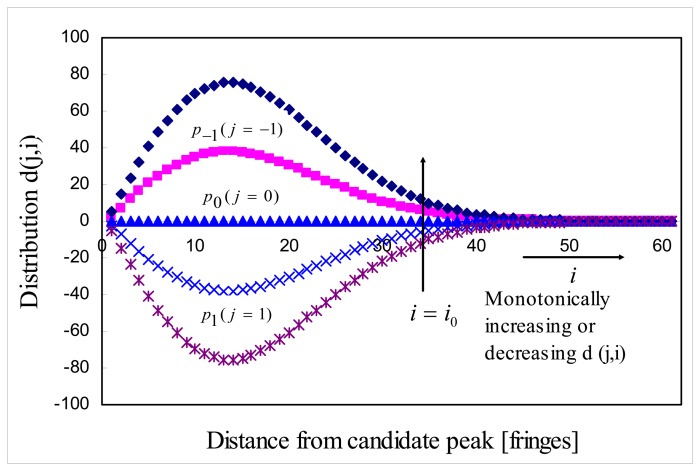
Example of distribution *d*( *j*, *i*) for noise free cross-correlation *i*(*n*).

Figure 3 is the example distribution *d*( *j*,*i*) of ideal noise-free cross-correlation *i*(*n*) . Finally, signal processing algorithm implements fine-tuning algorithm to obtain sub-sample resolution zero order fringe peak. As will be shown later, two-dimensional distribution *d*( *j*,*i*) is useful to analyze the data quality of fringe scans.

## 6. Experimental Results

### 6.1. Experimental arrangement

Experiments were carried out to demonstrate the performance of an all fiber white light interferometric absolute temperature sensor system. Before the measurement, interference fringe visibility was maximized adjusting polarization controllers in two arms of AFMZI (not shown in Figure 1). The whole system was stabilized for approximately four hours at room temperature after the system is turned on until no drifts of AFWLI signal are visually noticeable on the oscilloscope screen. During the tests SNR of the sensor system turned out to be approximately 50[dB]. SNR of the sensor system was calculated as follow.

After turning the system on *V_DC_* applied on the PZT1 was adjusted in order to make the path length difference between AFMZI and the sensor FFPI (and also the reference FFPI) larger than the coherence length of the light source. This adjustment eventually made the interference fringe totally disappeared on the oscilloscope screen. At this condition, noise voltage level of the reference FFPI and the sensing FFPI were 0.005 [volt]. and 0.008 [volt]. respectively when SLD forward current *I_F_*(SLD) = 80 [mA]. And peak-to-peak signal voltage level of the reference FFPI and the sensing FFPI of the interference fringe pattern were approximately 1.7 [volt] and 1.4 [volt] respectively during the sensor system operation. Then SNR of the reference FFPI and the sensing FFPI were calculated as 50 [dB] and 44 [dB], respectively.

### 6.2. Stability test

First, the stability of the proposed sensor system was tested to see if the sensor system calculates the same phase delay ΔΦ*^i^* repeatedly when the temperature of the sensing FFPI and the reference FFPI are fixed without any temperature control. For this purpose the sensing FFPI and the reference FFPI were placed together side-by-side (but not touching each other) between the styrofoam substrates enclosed by aluminum box so that both the sensing and the reference FFPIs are exposed to same room temperature. After approximately four hours of stabilization, 21 fringe scans were collected with a 10 minute separation. Sample rate was 36 [samples/fringe], PZT scanning period was 33 msec and fine tuning step was 1/1000 fringe. Fine tuning step is the signal processing parameter used in signal processing algorithm to enhance the accuracy of ΔΦ*^i^* . In stability test ΔΦ*^i^* was measured in term of total number of samples between $\Phi_{P,S}^{i}$ and $\Phi_{P,R}^{i}$. For example, if there are 36 samples in the range between $\Phi_{P,S}^{i}$ and $\Phi_{P,R}^{i}$, then ΔΦ*^i^* has the value of 36 samples which corresponds to *2π* phase delay (or one fringe) not considering the sign of phase delay. Table 1 shows the phase delay ΔΦ*^i^* between the sensing FFPI and the reference FFPI. In these measurements true phase delay ΔΦ*^i^* between sensing FFPI and reference FFPI was not known and the standard deviation of phase delay was calculated instead of root-mean-square error. Statistics in Table 1 showed that the proposed signal processing algorithm calculated the phase delays of 21 fringe scans with a standard deviation of 0.579 sample which is 0.015 fringe. Standard deviation of 0.015 fringe corresponds to 0.09 K of accuracy for a FFPI sensor of 10 mm cavity length [17].

### 6.3. Absolute temperature measurement

The second experiment shows the absolute temperature measurement. The temperature of the reference FFPI was maintained at 297K using a thermo-electric cooler (TEC) and the temperature of the sensing FFPI was varied from 297K to 300K using another TEC. Fringe scans were measured at the intervals of 1K for the sensing FFPI. Sample rate was 25 [samples/fringe] and PZT scan period was ∼9 second. Table 2 shows the mean and the standard deviation of temperature measurements in terms of sample. Five measurements were collected in a row at fixed sensing FFPI temperature. In this case the proposed signal processing algorithm repeatedly produced identical ΔΦ*^i^* at fixed sensing FFPI temperature. Note that fine tuning step was set as 1/1000 fringe and ±0.0005 fringe (half the fine tuning step) was the lowest obtainable fine tuning resolution. The resolution of approximately 0.0005 fringe was obtained with one exception that is the standard deviation 0.0001 fringe of five measurements (298K of sensing FFPI temperature) in Table 2.

**Figure 4.**
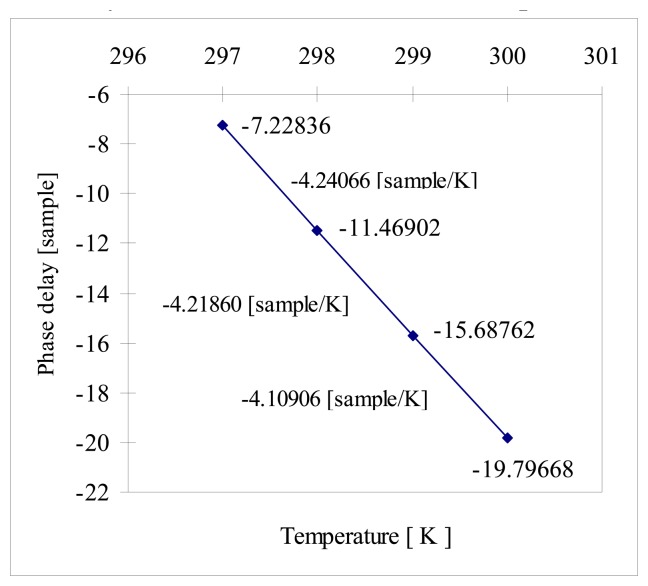
Phase delay ΔΦ*^i^* decrease in absolute temperature measurement.

Figure 4 shows the phase delay decrease between temperatures measurements. Note that difference of mean phase delay between temperature measurements (slope of straight line segment in Figure 4) was -4.24066, -4.21860, and -4.10906 samples/K.

Temperature dependence of these phase delays between temperature measurements was almost constant (linear) as assumed in Eq. (5) and Eq. (8). Slope was decreased by the amount of 0.13 samples (0.39 samples) over the 2K (6K or one fringe) temperature change. Sample rate was 25 [sample/fringe] and zero crossing period was shorten 1.5% over one fringe change. This was presumably attributed to the imperfect linear expansion of PZT. Note that in absolute temperature measurement test different set of reference FFPI and sensing FFPI (10mm cavity length) was used as the FFPIs used in the stability test were broken.

### 6.4. Hysteresis test

Practical application of the absolute temperature measurement sensor system must meet the wide range temperature measurement and also should not exhibit any hysteresis. In the hysteresis test the reference FFPI enclosed in the aluminum box was exposed to the room temperature without any temperature control. First, the sensing FFPI was inserted into a silica capillary tube free-standing to minimize the any friction applied to the sensing FFPI. The sensing FFPI in capillary tube and thermocouple were placed in the groove in the alumina block tip-to-tip ( but not touching, again) and the alumina block was placed in the oven. The resolution of thermocouple was 0.1K. The reference FFPI in the aluminum box was kept 3 feet away from the oven during the test. The oven was heated slowly from room temperature to 873K. Fringe scans were measured at intervals of 100K starting from 373K. The oven temperature was maintained at 873K for 10 minutes and cooled down to room temperature. Fringe scans were also measured during the cool-down at the same temperature at which fringe scans were measured while heating the oven.

Measured fringe scans were named starting with either “H” or “C”. “H” represents the heating-up and “C” represents the cooling-down. Comparison of zero order fringe peak at target temperature announced by proposed signal processing algorithm between heat-up and cool-down allows to see if the proposed sensor system, especially the sensing FFPI, is subject to any hysteresis. In the vicinity of the target temperature of heating and cooling process, control knob of oven was manually turned up and down simply because temperature-controlled oven was not available at the time of hysteresis test so that temperature of oven was varying 1K over 2 minutes during the PZT scanning and data acquisition. Three measurements were taken at one target temperature. Only the relatively good results are shown in Table 3. Time-lapse between two measurements at the target temperature was 10 minutes except four measurement were obtained in a row for ten minutes at 873K. In Table 3 phase delay ΔΦ*^i^* in terms of sample (bold and under-bar font) is the zero order fringe peak candidate *p_i_* (*i* = 0,±1,±2,±3,±4) the proposed signal processing algorithm announced as the zero order fringe peak. Fine tuning was not calculated in the hysteresis test.

As shown above, the hysteresis test of the sensor system worked successfully, except for a few exceptional fringe scan observations which drew our attention. The first observation in Table 3 is that identified zero order fringe peaks from two fringe measurements of heating process and cool-down process are not identical (fringe hopping). For example, *p*_0_ = –1109 for H373 but *p*_0_ = –1136 for C373.

**Figure 6.**
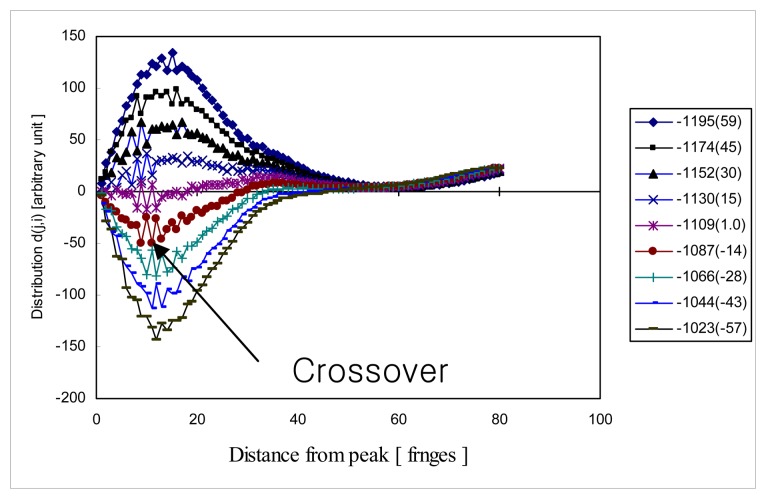
Distribution *d*(*j,i*) of fringe scan H373.

Figure 6 is the distribution *d*(*j,i*) of fringe scan H373. The legend of Figure 6 shows the zero order fringe peak candidate *p_i_* and its corresponding parameter *g*(*p_j_*) normalized with respect to the smallest *g*(*p_j_*). Some entries of *d*(*j*,*i*) for *p*_0_ = –1109 are noisy and have a crossover at which value of *d*(*j*,*i*) is changing from a negative to a positive value. This means that fringe scan is not as symmetric as the ideal case shown Figure 3, but the distribution *d*(*j*,*i*) for *p*_0_ = –1109 still shows the dominant candidate *p*_0_ = –1109 producing the smallest *g*(*p_j_*).

**Figure 7.**
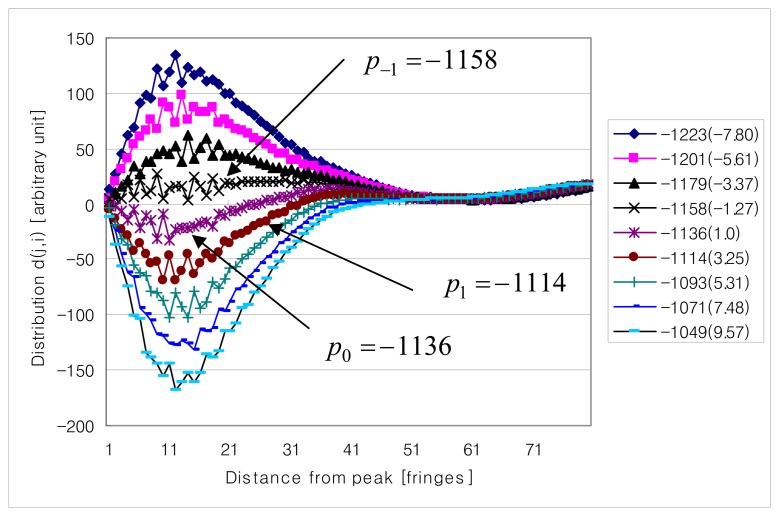
Distribution *d*( *j*,*i*) of fringe scan C373.

The second observation is that in Figure 7 for C373 one specific candidate is not dominant and *p*_0_ = –1136 and *p_-_*_1_ = –1158 in Figure 7 are competing each other for the smallest *g*(*p_j_*) (1.0 versus -1.27), contrary to Figure 6. In Table 3 it is shown that zero order fringe peak candidates *p_i_*'s of H373 are not identical to those of C373. This observation means that whole fringe scan itself shifted, for example, *p*_0_ = –1109 of H373 shifted to *p_-_*_2_ = –1179 of C373 or the visibility profile of the fringe scan (envelope shape of fringe scan) was changed.

But, possibly none of *p*_0_ = –1136 and *p*_-1_ = –1158 is the true zero order fringe peak and the true zero order fringe peak *p*_+1_ = –1114 shifted its position assuming *p*_0_ = –1109 of H373 is the same candidate as *p*_+1_ = –1114 of C373. Additionally, comparison of Figure 6 and Figure 7 shows that the envelope of the fringe scan (visibility profile of fringe scan) of H373 has changed to the envelope of the fringe scan of H373 during the test.

This property suggests that the proposed temperature sensing system exhibits the “fringe hopping” phenomenon and/or the hysteresis. The whole test took approximately 12 hours. The temperature of the reference FFPI was 297K when the hysteresis test was started and the reference temperature FFPI was increased to 298K by the time the hysteresis test was finished, presumably due to conduction heat from the oven. AFMZI and the reference FFPI were installed on the optical table side-by-side and AFMZI might also have been exposed to same conduction heat from the oven. This condition might have affected the hysteresis test unstable showing that phase delays are not identical for heating-up and cool-down (*p*_-1_= -686 for H473 but *p*_0_= −696 for C473). But, 1K temperature variation corresponds to 3∼4 [sample] for the case of 10 mm FFPI and must not be the reason for zero order fringe hopping from −1109 to −1136 (or from −686 to −696).

**Figure 8.**
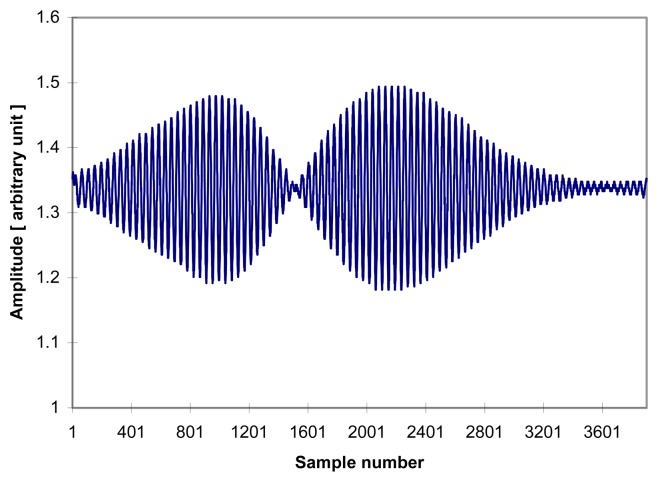
Example of bi-modal fringe scan (Sensing FFPI).

Many typical problems like unstable temperature stabilization of the aluminum box, light source temperature change (drift), bad sensors, PZT linearity and actual hysteresis characteristics of optical fiber might cause above mentioned phenomena which can be interpreted as the hysteresis. But, Morey [23] reported that optical fiber did not exhibit hysteresis unless fiber grating sensor were placed under a strain load at 923K more than 18 hours and as pointed out in reference [19] the author suspected that the birefringence modulation due to the bending and tension coiling of the 30 meter long fiber induced by the PZT voltage rather than hysteresis itself might cause polarization modulation, which will eventually induce a visibility change and fringe hopping judging from the “bi-modal” fringe scans (Figure 8) encountered during the hysteresis test

If the above projection is the case, then fiber becomes linearly birefringent [20, 21] and single mode fibers permit transmission of the two interfering orthogonal beams *I_x_* ( *x* – polarized) and *I_y_*(*y* – polarized). Assuming that birefringence induced by bending and tension coiling is:

(15) $$B_{\text{BTC}}=\beta_{x}-\beta_{y}$$

where *β_x_* and *β_y_* are the propagation constants of the *x*-polarized beam *I_x_* and *y*-polarized beam *I_y_*, the phase difference between two beams after going through AFMZI with its arm length of *L_P_* is given by:

(16) $$\Delta \Phi_{B}=B_{\text{BTC}}•L_{P}$$

Then the output signal is a superposition of two interference patterns *I_x_* and *I_y_* that might lead to poor contrast (envelope visibility change), fringe shifts [22] or bimodal fringe scan (fringe splitting in Figure 8) depending on the amount of phase difference ΔΦ*_B_*, whether the superposition of *I_x_* and *I_y_* is constructive or destructive, or the power splitting ratio between *I_x_* and *I_y_*.

The fringe scan in Figure 8 is one of the worst fringe scans obtained during the test period. Figure 8 is the fringe scan of the sensing FFPI in which ΔΦ*_B_* happened to be 26*λ* (approximate distance between two local maxima in Figure 8). This kind of fringe scan is termed a “bi-modal (fringe splitting)” fringe scan where the visibility profile has two local maxima while a “uni-modal” fringe scan is a fringe scan with only one global maximum (the zero order fringe peak), as shown in Figure 2. The fringe scan of the reference FFPI (not shown in this article) is also similar to Figure 8 and the cross-correlation fringe scan *i*(*n*) will not be symmetric at all.

During the hysteresis test the fringe scans on the oscilloscope screen were visually inspected and these bi-modal fringe scans were discarded, but if the degree of birefringence modulation is weak and two local fringe scans happen to be superimposed generating a fringe scan whose envelope is broadened in the vicinity of the zero order fringe peak and randomly changed, then broadening of fringe scan and/or random visibility change makes zero order peak identification easily prone to fringe hopping misidentification errors.

Additionally, the residual stress induced during the FFPI fabrication process using a fusion splicing causes birefringence, which can change the FFPI output along with the polarization status change (probably due to the bending and tension coiling of fiber mentioned above) of the input beam to the FFPI, so it is predicted that the combination of polarization modulation due to the birefringence of AFMZI and FFPI birefringence can cause a worse problem like the random change of envelope of fringe scan while heating or cooling, causing a fringe hopping from one fringe scan to another fringe scan even when obtained at the fixed target temperature. There has been speculation about the cause of fringe hopping, but this is still subject to more investigation. Replacing any possible source of fringe hopping one at a time should show which component is the dominant source of fringe hopping.

## 7. Robustness Test

Robustness is another requirement that the sensor system must meet. The author ran three more tests to check the robustness of the proposed sensor system: a light source temperature drift test, a PZT non-linearity test and a long term stability test. In the following tests (Sections 7.1. and 7.3) FFPI cavity length was 1 mm where one fringe corresponds to 60K and the sample rate was 36 samples/fringe.

### 7.1. Effect of laser diode temperature drift test

In order to achieve stable output power and lasing wavelength, it is crucial to control the temperature of the low coherence light source. Temperature drift of low coherence light source will usually induce a wavelength instability. Hence, the effect of laser diode temperature change on the measurement was tested by sweeping temperature of light source as follows.

A test similar to the stability test was used. First, the sensing FFPI and the reference FFPI were placed in the temperature shielded aluminum box and exposed to the room temperature without any temperature control. Then the temperature of the light source was varied from 285K to 303K by varying the voltage of temperature controller. The phase delay of fringe scans was measured at temperatures starting from 285K with 2K intervals. Maximum drift (difference between maximum phase delay and minimum delay among ten measurements) turned out to be 0.09 samples (0.0025 fringe) which corresponds to 0.15K (1 mm FFPI cavity) or 0.015K (10 mm cavity length FFPI). This value is not negligible, especially for high precision absolute temperature measurement. Therefore it is necessary that light source of the proposed sensor system be temperature-controlled for high precision absolute temperature measurement. But the proposed sensor system shows a medium-precision absolute temperature measurement capability against laser diode temperature drift.

### 7.2. PZT non-linearity test

Additionally, PZT can affect the robustness of the sensor system because PZT is only a moving (mechanical) component and it is sensitive to temperature drift, mechanical aging and non-linearity of PZT. The author couldn't test PZT's aging effect on the sensor system simply because a PZT aging acceleration tool was not available.

**Figure 9.**
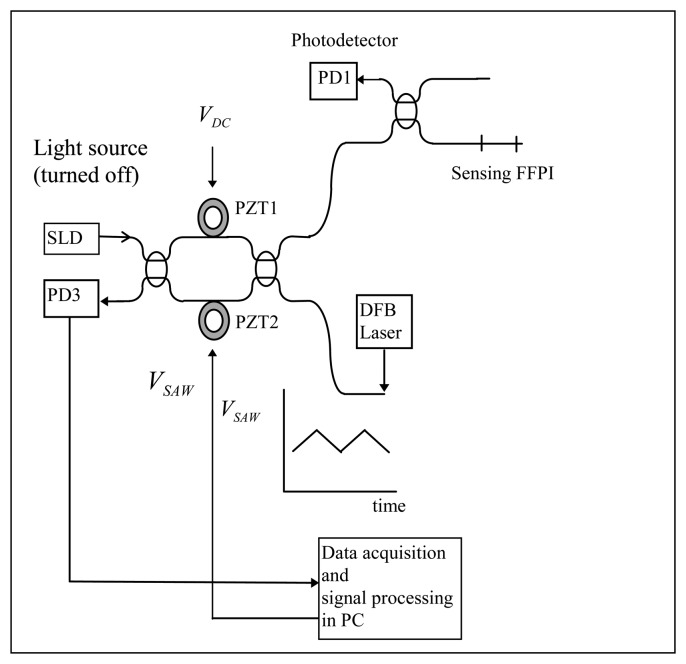
Configuration of set-up to measure non-linearity of scanning PZT.

To see the non-linearity of scanning PZT, AFWLI was modified to use a DFB laser as a light source as shown in Figure 9 and the SLD was turned off. As with AFWLI, the PZT2 of MZI was scanned so that the periodic interference of MZI is a function of PZT expansion. Then the changes of zero crossing period in the fringe scan were observed. The DFB laser has a long enough coherence length so that the periodic interference is observed through the whole PZT scanning range. First, 100 scans of fringe patterns *I_S_*(*n*) were collected. Then any D. C. component *I_S_*(*n*) was removed. After this zero crossing positions of *I_S_*(*n*) were calculated, for example, by applying the interpolation equation to two samples of *I_S_*(*n*) between which there is sign change. The sample rate was 22 samples/fringe and one scan has approximately 450 zero crossings. Two adjacent zero crossing made one zero crossing pair and the distance of each zero crossing pair was calculated.

**Figure 10.**
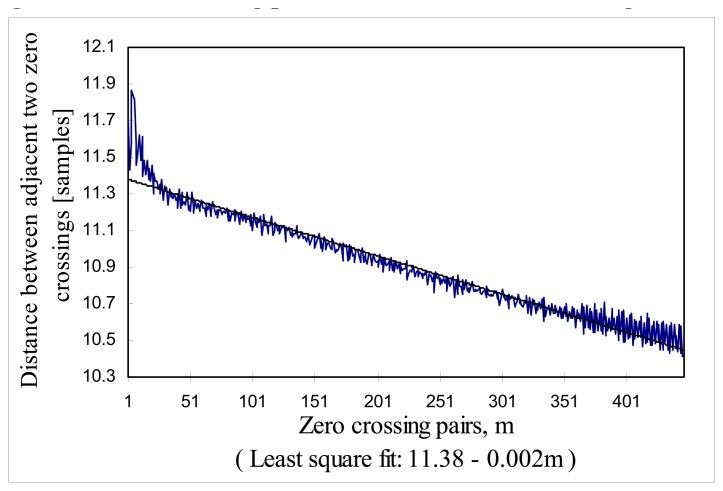
Zero crossing period decrease in PZT scanning window.

The distance of *m*-th zero crossing pair (*m*=1, 2,..,450) of the *j*-th ( *j*=1∼100) scan was stored in *Z*(*m,j*). The average distance of the *m*–*th* zero-crossing pairs of 100 scans were stored in *Z_avg_*(*m*). Figure 10 shows the zero crossing period changes of 450 zero crossing pairs, *Z_avg_*(*m*).

The straight line in Figure 10 is the least squares fit expression (-0.002*m+11.38) of zero crossing period as a function of zero-crossing pair number, *m*. This means that zero crossing period was reduced by the amount of 0.002 sample over half fringe. Sample rate was 22 samples/fringe and the zero crossing period was reduced by 0.018 % over one fringe change. This reduction is not quite consistent with the 1.5 % reduction mentioned in Section 6.3, but the reduction averaged over 100 scans measured with DFB laser light source is expected to be more accurate.

This result states that temperature measurement resolution of the proposed AFWLI will decrease towards one side of the PZT scanning window due to the non-linear PZT expansion, but this is not a critical adverse effect on the zero order fringe peak identification in that PZT non-linearity induces 1.8% of zero crossing period reduction over the scanning range of 100 fringes (600K dynamic range) and the zero order fringe peak was not missed during the stability test and absolute temperature measurement test shown above.

### 7.3. Effect of AFMZI temperature drift test

Robustness of a sensor system also requires that the sensor system show that the effects of AFMZI temperature drift and PZT temperature drift (sensitivity) on measurement are as low as possible. In the proposed sensor system long term stability is directly related to the long term stability of the copper chamber simply because the AFMZI is housed inside the double copper chamber.

For the long term stability test the absolute temperature measurement test was repeated under the same test conditions, but for five days. Reference FFPI and sensing FFPI were placed in the temperature-shielded aluminum box and the box was exposed to room temperature.

Again, the double copper chamber inside which the AFMZI was housed, was temperature-controlled using a thermistor and a TEC. The temperature inside the copper chamber was also monitored by measuring the resistance of other thermistors inside the double copper chamber. The maximum temperature drift inside the double copper chamber was 0.001K while room temperature was fluctuated over 296K to 297K for five days. Five measurements of phase delay were taken every 12 hours for five days. Eleven average fringe scan phase delays, each of which is the average phase delay of five measurements, were produced over a five day test period. A maximum drift of 0.039 sample (0.001 fringe) was obtained, which corresponds to 0.06 K (1 mm cavity length FFPI) or 0.006 K (10 mm cavity length FFPI).

In the above test it is intuitively clear that a 0.001 K drift of double copper chamber temperature will affect the long term stability of the proposed sensor system, but it is not easy to tell if the maximum drift of 0.039 sample can be mainly attributed to any long term instability of the PZT temperature drift or to a long term temperature drift of the 30 meter long fiber. It is speculated that the effect of temperature drift of the 30 meter long fiber is greater than the effect of PZT temperature drift, but further research is still needed to pinpoint the reason for the observed long term temperature drift instability of the double copper chamber.

## 8. Conclusions

In 1994 Kaddu demonstrated the WLI absolute temperature measurement system over the temperature range of 293K ∼ 343K, but in that scheme a Fabry-Perot type scanning interferometer was formed by a cleaved end of single mode fiber and a planar mirror, which was driven by a computer-controlled Nanomover (micro-positioning system) [18]. The feasibility of absolute temperature measurement using all fiber white light interferometry was demonstrated in this article. Combining an all fiber white light interferometer and a high resolution signal processing algorithm this sensor system can measure absolute temperatures up to 873K. In the stability test, signal processing algorithm repeatedly produced identical ΔΦ*^i^* with a resolution of 0.015 fringe (0.09K) at the fixed sensing FFPI temperature. For the absolute temperature measurement test, a thermocouple could not be used as the performance reference because the FFPI has a higher resolution than the thermocouple, but the sensor system showed that it was capable of obtaining 0.0005 fringe resolution (0.003K) which is consistent with the performance prediction of the signal processing algorithm. In the hysteresis test it seems like that the sensor system does not show any hysteresis, but birefringence modulation seems to cause fringe hopping. The system performance of a WLI sensing system not only depends on the interferometer hardware configuration itself, but also relies heavily on the signal processing algorithm used. In this article some speculation on the effect of birefringence and polarization modulation of AFMZI on the proposed sensor system was made, but these speculations are still subject to further investigation. Using polarization-insensitive components like an extrinsic air-cavity FFPI, a polarization-maintaining fiber Fabry-Perot interferometer or using a polarimetic interferometer as a processing interferometer in AFMZI is expected to help locate the source of misidentification. Also, the crossover of distribution *d*(*j,i*) is the result of the non-symmetric property of fringe scans. This property states that smallest value of the quality factor *g*(*p_j_*) is not a necessary and sufficient condition for any candidate peak to be a zero order fringe peak. Any signal processing algorithm needs to have error-detection or error correction capability to avoid misidentification due to fringe hopping induced by the hardware configuration.

Robustness of the proposed sensor system was tested using a laser diode temperature drift test, a PZT non-linearity test and an AFMZI temperature drift test. The PZT non-linearity test turned out not to affect the performance of the sensor system, and the two other tests showed that laser diode temperature drift affected the performance of the sensor system more than AFMZI temperature drift did, but in the context of a whole system view, it was shown that the proposed sensor system was robust against laser diode temperature drift and AFMZI temperature drift (double copper chamber temperature drift) for medium-precision absolute temperature measurements (<0.025 fringe). Comprehensive test of robustness requires more tests like effects of PZT aging, individual component temperature sweep and systematic polarization analysis. These tests will be the subject of future research.

## Figures and Tables

**Figure 5.**
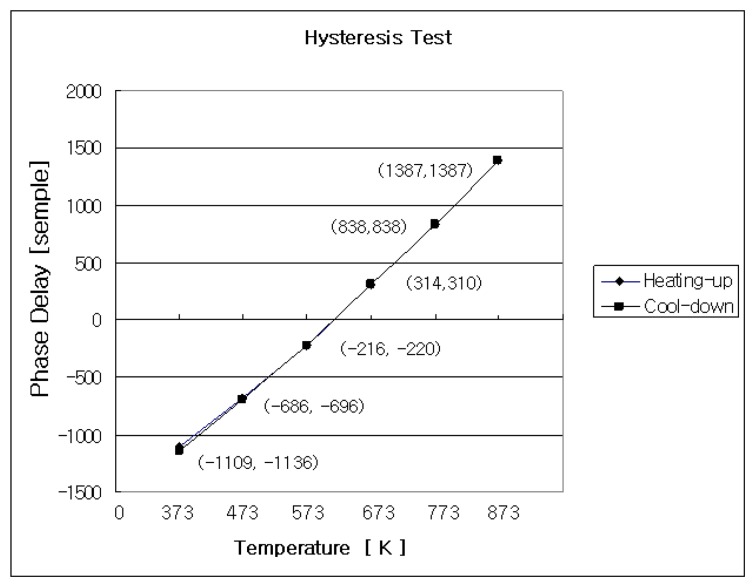
Hysteresis Test.

**Table 1.** Phase Delay ΔΦ*^i^* of stability test.

**Fringe scan number**	**Phase delay [sample]**	**Statistics [sample]**
1	54.10853	
2	54.14474	
3	54.10854	
4	54.07233	
5	54.03616	
6	53.89157	
7	53.78320	
8	55.18052	
9	53.56683	
10	53.56690	Mean:
11	53.53082	53.76878
12	53.46905	Standard Dev.;
13	53.36068	0.579
14	53.28855	(0.015 fringe)
15	53.18020	
16	53.14413	
17	53.14413	
18	54.71174	
19	54.60360	
20	53.14407	
21	53.10804	

**Table 2.** Phase delay ΔΦ*^i^* of absolute temperature measurement.

**Sensing FFPI Temp.** **[ K ]**	**Phase delay** **[sample]**	**Statistics** **[sample]**
297	-7.223328	Mean:
297	-7.248166	-7.22836
297	-7.223475	Standard Dev.:
297	-7.223486	0.0110
297	-7.223417	(0.0004 fringe)
298	-11.47066	Mean:
298	-11.47117	-11.46902
298	-11.47101	Standard Dev.:
298	-11.47105	0.0043
298	-11.46120	(0.0001 fringe)
299	-15.70248	Mean:
299	-15.70258	-15.68762
299	-15.67775	Standard Dev.:
299	-15.67764	0.0136
299	-15.67766	(0.0005 fringe)
300	-19.80177	Mean:
300	-19.80181	-19.79668
300	-19.77718	Standard Dev.:
300	-19.80161	0.0109
300	-19.80165	(0.0004 fringe)

**Table 3.** Hysteresis Test.

**Heat-up**				**Cool-down**			
**Temp.** **[ K ]**	**Zero-Order Fringe Peak Candidates [samples]**			**Zero-Order Fringe Peak Candidates** **[samples]**			**Temp.** **[ K ]**
H373	*p*_−1_ -1130	*p*_0_ **-1109**	*p*_+1_ -1087	*p*_−2_ -1179	*p*_−1_ -1158	*p*_0_ **-1136**	C373
H473	*p*_−2_ -708	*p*_−1_ **-686**	*p*_−0_ -665	*p*_−1_ -718	*p*_0_ **-696**	*p*_+1_ -675	C473
H573	*p*_−1_ -237	*p*_0_ **-216**	*p*_+1_ -195	*p*_−1_ -241	*p*_0_ **-220**	*p*_+1_ -189	C573
H673	*p*_−1_ 292	*p*_0_ **314**	*p*_+1_ 335	*p*_−2_ 288	*p*_−1_ **310**	*p*_0_ 331	C673
H773	*p*_0_ 817	*p*_+1_ **838**	*p*_+2_ 860	*p*_−1_ 817	*p*_0_ **838**	*p*_+1_ 859	C773
873-1st	*p*_−1_ 1366	*p*_0_ **1387**	*p*_+1_ 1409	*p*_0_ 1366	*p*_+1_ **1387**	*p*_+2_ 1409	873-3rd
